# A biological extract of turmeric (*Curcuma longa*) modulates response of cartilage explants to lipopolysaccharide

**DOI:** 10.1186/s12906-019-2660-z

**Published:** 2019-09-11

**Authors:** Wendy Pearson, Laima S. Kott

**Affiliations:** 1 Department of Animal Biosciences, University of Guelph, Guelph, ON N1G 2W1 Canada; 2 Department of Plant Agriculture, University of Guelph, Guelph, ON Canada

**Keywords:** Arthritis, Cartilage inflammation, Simulated digestion, Hepatic metabolism, Turmeric

## Abstract

**Background:**

Turmeric is commonly used as a dietary treatment for inflammation, but few studies have evaluated the direct effect of turmeric on cartilage. The purpose of this study was to characterize cartilage explants’ inflammatory responses to lipopolysaccharide in the presence of a simulated biological extract of turmeric.

**Methods:**

Turmeric was incubated in simulated gastric and intestinal fluid, followed by inclusion of liver microsomes and NADPH. The resulting extract (TUR_sim_) was used to condition cartilage explants in the presence or absence of lipopolysaccharide. Explants were cultured for 96 h (h); the first 24 h in basal tissue culture media and the remaining 72 h in basal tissue culture media containing TUR_sim_ (0, 3, 9 or 15 μg/mL). Lipopolysaccharide (0 or 5 μg/mL) was added for the final 48 H*. media* samples were collected immediately prior to lipopolysaccharide exposure (0 h) and then at 24 and 48 h after, and analyzed for prostaglandin E_2_ (PGE_2_), glycosaminoglycan (GAG), and nitric oxide (NO). Explants were stained with calcein-AM for an estimate of live cells. Data were analyzed using a 2-way repeated measures (GAG, PGE_2_, NO) or 1-way ANOVA without repeated measures (viability). Significance accepted at *p* < 0.05.

**Results:**

TUR_sim_ significantly reduced PGE_2,_ NO and GAG, and calcein fluorescence was reduced. Conclusions: These data contribute to the growing body of evidence for the utility of turmeric as an intervention for cartilage inflammation.

## Background

Turmeric (*Curcuma longa*) is a South Asian perennial herb of the ginger family. Contemporary research providing evidence for the anti-inflammatory and anti-arthritic effects of turmeric first appeared in the scientific literature almost half a century ago [1, 2]. Since then more than 1100 research studies have been documented which describe anti-inflammatory effects of turmeric and/or its principle bioactive curcumin. Several recent reviews of the literature pertaining to turmeric and/or curcumin [3–6] unanimously conclude that the research evidence supports their systemic anti-inflammatory and anti-arthritic effects. There are, however, few studies that explore direct effects of turmeric on cartilage independent of systemic anti-inflammatory activity. These studies are important as they serve to illuminate mechanism of anti-arthritic action on the target tissue. Two studies have reported effects of curcumin on equine [7] and human [8] cartilage explants. Conditioning of equine cartilage explants with curcumin (100 μmol/L) for 5 days significantly reduced interleukin-(IL-)1β-induced release of glycosaminoglycan (GAG; a measure of cartilage breakdown) [7]. This inhibition of GAG release was not observed in human cartilage explants, perhaps due to a lower curcumin exposure rate (5–20 μmol/L), but these authors did report an inhibition of IL-1-induced nitric oxide (NO), prostaglandin E_2_ (PGE_2_), IL-6, IL-8 and matrix metalloproteinase 3 [8]. While these 2 studies provide rationale for further investigating direct effects of curcumin on cartilage, their usefulness is limited by the fact that neither study accounted for the fact that curcumin is in most cases provided orally. This oral route of administration exposes curcumin to enzymatic and pH-mediated alteration, the effects of which are not accounted for in the previous 2 studies. Furthermore, the effects of turmeric (the parent material of curcumin) on cartilage explants has not been investigated.

It is hypothesized that turmeric reduces inflammation in cartilage, and this effect is not muted by the effects of gastrointestinal digestion or hepatic metabolism. The purpose of the current experiment was to evaluate direct anti-inflammatory effects of a simulated biological extract of turmeric on cartilage explants.

## Methods

All chemical reagents were purchased from Sigma Aldrich Canada (Oakville ON) unless otherwise indicated. All spectrophotometric and fluorescence analyses were conducted using a 1420 Victor 2 spectrophotometer (Perkin Elmer; Woodbridge ON). Turmeric was purchased from Amoros Nature, S.L. Hostaliric-Girona, Spain (Lot number 16.01744; Certificate of Analysis provided in Supplementary Files).

### Biological extraction

A biological extract of turmeric was prepared in a manner to simulate upper gastrointestinal digestion (stomach and small intestine) and first pass biotransformation by the liver, as previously described [9]. Briefly, turmeric (0.10 g) (Selected Bioproducts Inc. Guelph ON) was incubated on a tube rocker in 15 mL of simulated gastric fluid (37 mM NaCl, 0.03 N HCl, 3.2 mg/mL pepsin) at 37 °C for 2 h. Subsequently, pH was adjusted to 7.0 and an equal volume of simulated intestinal fluid (30 mM K_2_HPO_4_, 160 mM NaH_2_PO_4,_ 20 mg/mL pancreatin) was added, and the mixture was incubated on a tube rocker at 37 °C for an additional 2 h. Liver microsomes (from male rat, purchased from Sigma Aldrich; catalogue number M9066) and NADPH were then added, and the mixture was incubated for an additional 30 min. Finally the mixture was centrifuged and the supernatant filtered through a 50 kDa centrifuge filtration unit (Amicon Ultra) and refrigerated until use.

### Cartilage explants

Front limbs from 8 pigs were obtained post-mortem from a federally-inspected meat processing facility within 1 h of humane slaughter for the purpose of human food. Limbs were transported on ice to the laboratory, the intercarpal joint was exposed, and explants were aseptically excised from both articulating surfaces using a 4 mm dermal biopsy tool. Explants were arranged (2 per well) in 24-well tissue culture plates containing 1000 μL/well of DMEM supplemented with amino acids, sodium bicarbonate, pen/strep, glutamine, ascorbic acid and 10% FBS as previously described [9]. Media was removed and refreshed from wells every 24 h for a total culture duration of 96 h.

After the first 24 h of culture, and for each day thereafter, TUR_sim_ was added to wells at a rate of 0, 3, 9, or 15 μg/mL such that tissue from each animal was exposed to each dose. Assuming a total body water fluid volume of 45% (36 L in a 80 kg human), these inclusion rates approximate a human bioavailable turmeric dose of 108, 324 and 540 mg, respectively. During the final 48 h of culture, an inflammatory stimulus [lipopolysaccharide (LPS)] (0 or 5 μg/mL) was added to the wells.

### Sample analysis

Media samples from the final 48 h of culture were analyzed for PGE_2_, GAG, and NO (Griess reaction). Immediately following the final collection of media, explants were placed into 96-well micro-titre plates and incubated with 200 μL of 4 mM calcein for 40 min for an estimate of viability of chondrocytes within explants. Calcein fluorescence was obtained using excitation/emission filters of 485/530 nm.

Media PGE_2_ concentrations were determined using a commercially-available ELISA kit (R&D Systems; Cat # KGE004B) according to manufacturer instructions. A best-fit 3rd order polynomial standard curve was developed for each plate (R^2^ ≥ 0.99), and these equations were used to calculate PGE_2_ concentrations for samples from each plate.

GAG concentrations were determined with the 1,9-dimethyl methylene blue (DMB) staining assay. Samples were added to 96-well microtitre plates at 50% dilution, then serially diluted 1:2 up to a final dilution of 1:64. Guanidine hydrochloride (275 mg/mL) was added to each well, followed immediately by addition of 150 μL DMB reagent. Absorbance was measured at 530 nm. Sample absorbance was compared to that of a bovine chondroitin sulfate standard. A best-fit linear standard curve was developed for each plate and these equations were used to calculate GAG concentrations for samples.

Nitrite (NO^2−^), a stable oxidation product of NO, was analyzed by the Griess Reaction. Undiluted samples were added to 96-well microtitre plates. Sulfanilamide (0.01 g/mL) and N-(1)-Napthylethylene diamine hydrochloride (1 mg/mL) dissolved in phosphoric acid (0.085 g/L) was added to all wells, and absorbance was read within 5 min and 530 nm. Sample absorbance was compared to a sodium nitrite standard. A best-fit linear standard curve was developed for each plate, and these equations were used to calculate nitrite concentrations for samples.

### Data analysis

Data are presented as means ± SEM unless otherwise noted. Time ‘0 h’ represents the media sample obtained immediately prior to inclusion of LPS, while time 24 and 48 h represent media samples 24 and 48 h after exposure to LPS. PGE_2_, GAG and NO data were analyzed using a 2-way repeated measures (with respect to treatment and time) ANOVA. Viability data were analyzed using a one-way ANOVA. When a significant F-ratio was obtained the Holm-Sidak post-hoc test was used to identify significantly different means. Significance was accepted when *p* < 0.05.

## Results

### PGE_2_

**Fig. 1:**
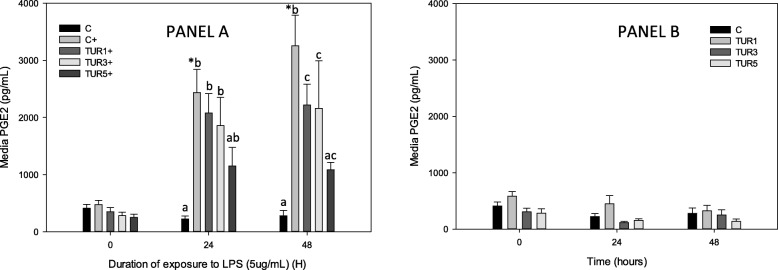
PGE_2_ production by cartilage explants (*n* = 8 pigs) conditioned with simulated biological extract of turmeric (3, 9 or 15 μg/mL; TUR1, TUR3 and TUR5 respectively) compared with simulated biological extract containing no turmeric (C) in the presence (Panel **a**) or absence (Panel **b**) of lipopolysaccharide (LPS; 5 μg/mL) (+).Explants were cultured for a total of 96 h and were conditioned with TUR for the first 48 h prior to exposure to LPS. Data are shown for the final 48 h only. * denotes significant change from baseline within treatments. Letters denote significant differences between treatments at specific time points

Stimulation of control explants with LPS produced a significant increase in PGE_2_ at 24 and 48 h (Fig. 1a and b). Conditioning of explants with TUR_sim_ (15 μg/mL) significantly reduced LPS-induced PGE_2_ at 24 and 48 h. TUR_sim_ (3 and 9 μg/mL) also significantly inhibited PGE_2_ production at 48 h (Fig. 1a). Conditioning with TUR_sim_ did not affect PGE_2_ production in explants not stimulated with LPS (Fig. 1b).

### GAG

**Fig. 2:**
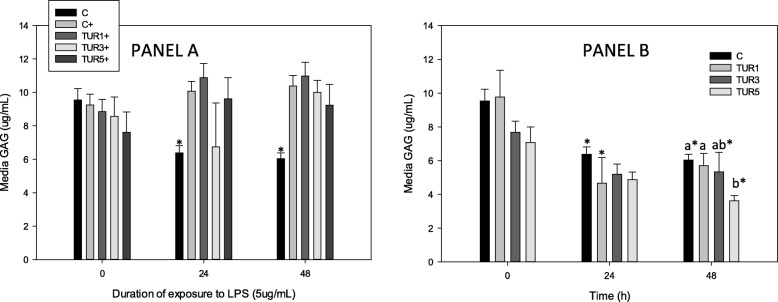
GAG production by cartilage explants (*n* = 8 pigs) conditioned with simulated biological extract of turmeric (3, 9 or 15 μg/mL; TUR1, TUR3 and TUR5 respectively) compared with simulated biological extract containing no turmeric (C) in the presence (Panel **a**) or absence (Panel **b**) of lipopolysaccharide (LPS; 5 μg/mL) (+). Explants were cultured for a total of 96 h and were conditioned with TUR for the first 48 h prior to exposure to LPS. Data are shown for the final 48 h only. * denotes significant change from baseline within treatments. Letters denote significant differences between treatments at specific time points

Stimulation of control explants with LPS produced a significant increase in GAG at 24 and 48 h (Fig. 2a and b) compared with unstimulated controls (Fig. 2a). In explants not stimulated with LPS, TUR_sim_ (9 and 15 μg/mL) significantly reduced overall GAG compared with unstimulated controls; media GAG was significantly lower in unstimulated explants conditioned with TUR_sim_ (15 μg/mL) at 48 h compared with unstimulated controls (Fig. 2b).

### NO

**Fig. 3:**
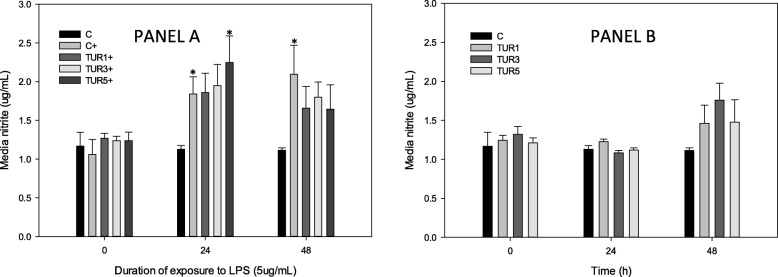
NO production by cartilage explants conditioned with simulated biological extract of turmeric (3, 9 or 15 μg/mL; TUR1, TUR3 and TUR5 respectively) compared with simulated biological extract containing no turmeric (C) in the presence (Panel **a**) or absence (Panel **b**) of lipopolysaccharide (LPS; 5 μg/mL). Explants were cultured for a total of 96 h and were conditioned with TUR for the first 48 h prior to exposure to LPS. Data are shown for the final 48 h only. * denotes significant change from baseline

Stimulation of control explants with LPS (5 μg/mL) produced a significant increase in NO at 24 and 48 h (Fig. 3a). This increase was not seen in explants conditioned with TUR_sim_ (3 and 9 μg/mL), and TUR_sim_ (15 μg/mL) prevented increase in LPS-induced NO production only at 48 h. There were no significant differences between any dose of TUR_sim_ and stimulated control explants at any time point.

### Viability

**Fig. 4:**
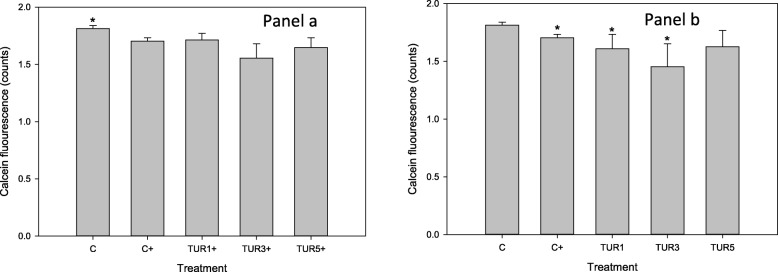
Calcein staining (as a measure of cell viability) of cartilage explants conditioned with simulated biological extract of turmeric (3, 9 or 15 μg/mL; TUR1, TUR3 and TUR5 respectively) compared with simulated biological extract containing no turmeric (C) in the presence (Panel **a**) or absence (Panel **b**) of lipopolysaccharide (LPS; 5 μg/mL). Explants were cultured for a total of 96 h and were conditioned with TUR for the first 48 h prior to exposure to LPS. * denotes significant change from baseline

Stimulation of control explants with LPS (5 μg/mL) for 48 h resulted in a significant (*p* = 0.01) decline in calcein fluorescence (a measure of cell viability) (Fig. 4). There was no difference in calcein flourescence amongst all LPS-stimulated explants irrespective of exposure to TUR_sim_ (Fig. 4a). However, in unstimulated explants TUR_sim_-conditioning (3 and 9 μg/m) resulted in a significant decline in viability (Fig. 4).

## Discussion

The main findings of this study are that cartilage explants, when exposed to a biological extract of turmeric in the presence of LPS, produced various reductions in PGE_2_, GAG and NO compared with LPS-stimulated controls. Furthermore, unstimulated explants had reduced GAG loss and reduced staining with calcein-AM.

In the current study we utilized methodology which provides a reasonable approximation for the effects of upper gastrointestinal digestion, hepatic metabolism and diffusion across the synovial membrane. Simulated digestion (gastric and intestinal) is an important consideration during in vitro evaluation of products or compounds that are typically consumed orally. It accommodates, at least in part, the effects of changing pH and enzymatic degradation on potentially bioactive compounds during digestion which may alter their effects on target tissues [10–12]. Furthermore, biotransformation and bioactivation by the liver can profoundly influence effects of dietary products on target tissues, and can be a serious limitation to extrapolating in vitro data to in vivo situations [13]. We have previously demonstrated that the simulated hepatic metabolism step utilized in the current study effectively contributes to formation of 3 known post-hepatic biotransformation products of rosmarinic acid [9]. Thus we employed this methodology to trigger liver metabolism of potentially bioactive compounds within turmeric in the current study, and TUR_sim_ strongly inhibited LPS-induced PGE_2_ in a dose-dependent manner. While curcumin is generally considered to be the bioactive compound in turmeric [14, 15], it undergoes extensive metabolism in the liver, producing curcumin glucuronide, curcumin sulfate, tetrahydrocurcumin, hexahydrocurcumin, octahydrocurcumin, and hexahydrocurcuminol [16, 17]. Our observed inhibition of LPS-induced PGE_2_ by TUR_sim_ is consistent with curcumin-induced inhibition of PGE_2_ production by human chondrocytes and human cartilage explants [8]. However, others have demonstrated that curcumin biotransformation products (tetrahydrocurcumin, curcumin sulfate and hexahydrocurcumin) have only a weak inhibitory effect on phorbol ester-induced PGE_2_ production in vivo [16], suggesting that post-hepatic curcumin is not the only product of turmeric digestion and metabolism contributing to our observed inhibition of PGE_2_. We did not characterize the post-hepatic species in our biological extract for the current study, and future research should attempt to identify the major phytochemical parents and downstream metabolic endproducts in TUR_sim_ to further define those with inhibitory effects on PGE_2_.

The slight inhibitory effect of TUR_sim_ (15 μg/mL) on LPS-induced cartilage breakdown (as measured by increased release of GAG into tissue culture media) demonstrated in the current study is less marked than that reported for curcumin in IL-1-stimulated equine cartilage explants [7], perhaps due (at least in part) to the very high concentration of curcumin employed in the latter study (100 μM, equivalent to 36.8 μg/mL). Assuming a curcumin concentration of approximately 3% in turmeric powder [18], a curcumin concentration of 36.8 μg/mL would be represented in a turmeric concentration of about 1227 μg/mL – markedly higher than the concentration of TUR_sim_ in the current study. The GAG inhibition observed at our highest dose occurred subsequent to an already-blunted GAG concentration prior to exposure to LPS, likely resulting from the 48 h of exposure to TUR_sim_ prior to stimulation with LPS. In unstimulated explants, the inhibition of GAG release was significant at the intermediate (9 μg/mL) and highest (15 μg/mL) doses of TUR_sim_. This is consistent with others [8] who report that curcumin (5–20 μM, equivalent to 1.8–7.4 μg/mL) did not significantly inhibit IL-1-induced GAG release from human cartilage explants, but did reduce GAG loss in unstimulated explants. This result may arise, at least in part, from a reported inhibitory effect of curcumin on matrix metalloproteinases that facilitate disassembly of matrix proteoglycans [5]. The effect of post-hepatic transformation products of turmeric and/or its bioactive principles on GAG release from cartilage has not been previously reported and should be explored in future research.

The effects of turmeric and curcumin on NO are unclear. In the current study, TUR_sim_ prevented a significant stimulatory effect of LPS on NO production, but did not result in significantly lower NO than in stimulated control explants at any point. This effect appears to result from a slightly (non-significantly) elevated media NO prior to stimulation with LPS, and does not provide support for an inhibitory effect of TUR_sim_ on NO production. The IC_50_ of an ethanolic extract of *C. aromatica* on LPS-induced NO production in murine macrophages is 12.27 μg/mL [19]. Using the same model, others report an IC_50_ of 23.4 μg/mL of a methanolic extract of *C. zedoaria* [20]. These exposure rates are in reasonable agreement with that of the current study, but we perhaps did not observe this effect because the biological extraction procedure modified the bioactivity of our test material. Also, the authors of the former studies utilized a different varieties of turmeric than that used in the current study, and the extracts were applied to macrophages rather than cartilage explants; all these factors may have contributed to the differing results of the current study. Others have explored the inhibitory effect of a water extract of *C. longa* on murine macrophages [21], and report that significant inhibition of LPS-induced NO was not measured until exposure rates exceeding 150 μg/mL – a 10-fold higher exposure rate than the highest rate in the current study. A methanolic and ethyl acetate extract inhibited NO at exposure rates of 25 and 6.25 μg/mL (respectively) [21]. There are no studies reporting effects of *C. longa* on production of LPS-induced NO in cartilage explants with which to compare our data, and no studies which seek to identify post-hepatic biotransformation products in this model. However, our data support a mild inhibitory effect of TUR_sim_ on LPS-induced NO production which may contribute to the anti-arthritic effect reported from turmeric [22, 23], curcumin [24–26], and other turmeric-based compounds [27–29].

.Calcein fluorescence is a well-established technique for comparing populations of live cells in a variety of tissues including arterial explants [30] and cartilage explants [31]. The significant decline in calcein fluorescence in unstimulated explants exposed to TUR_sim_ in the current study was not expected, and is contrary to evidence that curcumin protects against IL-1-induced cell death in primary chondrocytes [32–34]. Thus, we do not have a strong basis from which to conclude that our observed decline in fluorescence indicates promotion of cell death. An alternative explanation may be found in the well-documented stimulatory effect of curcumin [35–39] and its post-hepatic metabolite tetrahydrocurcumin [40] on autophagy. Autophagy is a physiological process which facilitates organized disassembly of damaged proteins and organelles in order to preserve energy balance and tissue homeostasis [41]. Non-selective autophagy plays a central role in bulk turnover of cytoplasm and sequesters various compounds that can influence calcein fluorescence into lysosomes. This has been demonstrated in HeLa cells for which calcein-AM was utilized to estimate cytosolic labile iron; autophagic degradation of ferruginous material resulted in sequestering of labile iron into lysosomes, and subsequent decrease in calcein fluorescence (and underestimate of intracellular labile iron) [42]. It is also plausible that TUR_sim_ may have contributed to decline in calcein fluorescence by promotion of cytotoxicity, as autophagy is a well-known mechanism for cell death under some circumstances [42, 43]. It not known if augmented autophagy contributed to the observed decline in calcein fluorescence in the current study, but this should be investigated in future research. Furthermore, future studies should also include an estimate of non-viable cells [eg. Staining of non-viable cells with ethidium homodimer-1] [9] and/or apoptosis [TUNEL assay] [44].

The methods used to test our hypothesis were designed to improve upon more conventional extraction methods for in vitro studies by incorporating effects of gastrointestinal digestion, hepatic metabolism, and diffusion across biological membranes. In addition to the limitations previously acknowledged, these methods are also limited by the cross-species use of cartilage from swine, and liver microsomes from rat. Porcine articular cartilage is significantly thinner (1.2 vs 1.8 mm, respectively) and more permeable (6.3 vs 2.0 × 10^− 16^ m^4^/Ns, respectively) than human cartilage [45]. Thus results reported herein may differ from that which might be observed with human cartilage. Furthermore, rat liver microsomes contain approximately 50% of the amount of lipid per mg protein than human liver microsomes [46], and enzymatic activity of microsomes have long been known to differ with developmental age [47] and species [47, 48]. There are also notable differences between rat and human microsomal metabolism of compounds [32] which may also influence interpretation of our results within the context of human inflammation. The extent to which these limitations influence interpretation of data in the current study should be explored in future in vivo studies. These studies should also seek to validate the simulated digestion and biotransformation of oral turmeric by quantifying the levels and distribution of its active components in blood and articulating joints of pre-clinical animal models. In addition, the results reported herein are in response to LPS, and not to the endogenous pro-arthritis stimulus of interleukin-1β (IL-1) [49]. While IL-1 undoubtedly participates in the complex catabolic and pro-inflammatory signaling in OA, a recent review has concluded its contention as the prototypical catalyst for disease initiation and progression is in decline [50]. However, it may be of value to conduct further studies using IL-1 as the pro-inflammatory stimulus in order to compare cartilage responses to those reported in the current study.

## Conclusion

A biological extract of turmeric reduces inflammatory responses of cartilage to LPS and contributes to the literary evidence for use of the spice to reduce articular inflammation and catabolism. The reason for decline in calcein fluorescence in TUR_sim_-exposed cartilage explants is not known, but should be explored in further research.

## Data Availability

The datasets used and/or analysed during the current study are available from the corresponding author on reasonable request.

## Abbreviations

GAG: Glycosaminoglycan
h: Hours
IL: Interleukin
LPS: Lipopolysaccharide
NADPH: Nicotinamide adenine dinucleotide
NO: Nitric oxide
PGE_2_: Prostaglandin E_2_
TUR_sim_: Simulated biological extract of turmeric
